# Validation of a new three-dimensional imaging system using comparative craniofacial anthropometry

**DOI:** 10.1186/s40902-017-0123-3

**Published:** 2017-08-25

**Authors:** Farhad B. Naini, Sarah Akram, Julia Kepinska, Umberto Garagiola, Fraser McDonald, David Wertheim

**Affiliations:** 1 Kingston and St George’s Hospitals and Medical School, London, UK; 2 King’s College London Dental Institute, London, UK; 3 Guy’s and St. Thomas’ Hospitals NHS Foundation Trust, London, UK; 4 Department of Reconstructive and Diagnostic Surgical Sciences, University of Milan, Milan, Italy; 5 King’s College London Dental Institute, London, UK; 6 Faculty of Science, Engineering and Computing, Kingston University, London, UK

**Keywords:** Three-dimensional imaging, Craniofacial, Anthropometry

## Abstract

**Background:**

The aim of this study is to validate a new three-dimensional craniofacial stereophotogrammetry imaging system (3dMDface) through comparison with manual facial surface anthropometry. The null hypothesis was that there is no difference between craniofacial measurements using anthropometry vs. the 3dMDface system.

**Methods:**

Facial images using the new 3dMDface system were taken from six randomly selected subjects, sitting in natural head position, on six separate occasions each 1 week apart, repeated twice at each sitting. Exclusion criteria were excess facial hair, facial piercings and undergoing current dentofacial treatment. 3dMDvultus software allowed facial landmarks to be marked and measurements recorded. The same measurements were taken using manual anthropometry, using soluble eyeliner to pinpoint landmarks, and sliding and spreading callipers and measuring tape to measure distances. The setting for the investigation was a dental teaching hospital and regional (secondary and tertiary care) cleft centre. The main outcome measure was comparison of the craniofacial measurements using the two aforementioned techniques.

**Results:**

The results showed good agreement between craniofacial measurements using the 3dMDface system compared with manual anthropometry. For all measurements, except chin height and labial fissure width, there was a greater variability with the manual method compared to 3D assessment. Overall, there was a significantly greater variability in manual compared with 3D assessments (*p* < 0.02).

**Conclusions:**

The 3dMDface system is validated for craniofacial measurements.

## Background

Facial aesthetic and reconstructive surgery, as a distinct clinical discipline, requires extensive planning, where photography forms a central role. Somewhat unique to cleft and craniofacial care is the prolonged treatment time, where patients can be in treatment for perhaps two decades. As a result, patient photographs need to be reproducible, and any image capture system must be validated for accuracy and reproducibility, with three-dimensional systems providing potentially the most useful diagnostic, planning, audit and research tools.

In craniofacial research, quantitative methods of measurement are important. The traditional direct method of facial surface anthropometry is non-invasive; however, it is time consuming, requires operator training for accurate results and may not be always practical in the clinical setting [1]. The ideal three-dimensional technology should provide quick and efficient image capture, which is consistently repeatable [2]. Three-dimensional imaging has the potential for accurate facial measurement and permits the clinician to take measurements in the absence of the patient once the image has been captured and virtually stored. It may also provide an invaluable interactive tool for discussion with patients when communicating existing problems and exhibiting more accurate outcomes [3].

The imaging technology may be used to observe the behaviour of the soft tissues more accurately compared to hand tracing and 2D photographic predictions. Such imaging science can also be utilised to assess growth-related facial changes [4]. As well as being non-invasive, it carries no risk to the patient in terms of radiation and no contact with the tissues ensures there is no distortion of the image, which may affect the measurements.

Practical benefits include ease of storage of image data, which can be transferred with ease between clinicians and hospitals. Moreover, it can provide data for auditing and research purposes [5].

The creation of the 3D image involves capturing the geometry of the face and the colour information, which is then applied to the underlying shape information. This is done through the following three distinct steps [6, 7]:

1. 3D surface capture: There are two ways this can be achieved, via either an optics-based approach or a laser approach.
2. Modelling: The physical properties of the face are expressed mathematically via complex algorithms. This creates a polygonal mesh (made up of small polygons). The area within the polygons is filled with the surface pixels.
3. Rendering: The colour information is incorporated to provide texture and depth and provide a lifelike 3D object to view.

Heimlich [8] aptly stated that “the eye can grasp an idea many times faster than the ear and generally retain it for longer”. Early 3D stereophotogrammeteric images were produced via a cartography-based technique, which acted as a precursor to the modern 3D imaging [9]. The patient was asked to lie supine with their head fixed in position, using a cephalostat type set-up [10]. However, modern techniques encompass two methods for creating a 3D surface image—laser based and optics based. Optics-based systems may use structured light, Moiré Fringe projection, or stereophotogrammetry [11–23].

Stereophotogrammetry has been reported as being superior to Moiré topography and structured light owing to instantaneous image capture, making it ideal for clinical use, particularly with children [14]. In addition, the set-up is relatively simple, with a short calibration process, and it produces a more complete and accurate 3D image, which can be manipulated to view all planes. It also permits the accurate location of various landmarks. The software is able to combine 3D images with CBCT and CT images, allowing a layer-by-layer examination of the patient. Furthermore, and perhaps most importantly, many of the stereophotogrammetric systems allow the subject to be orientated in a natural head position, with their eyes open [24].

The majority of validation-based experiments have used plaster models of the head to check reproducibility of landmarks. One investigation found that the landmarks requiring palpation on the face prior to image capture had poor reproducibility, compared to those, which could be landmarked without palpation. Overall, the system was found to be accurate within 0.4 mm and was advocated for recording cleft deformities and measuring changes following surgery [25]. However, the noted limitations of this earlier system were the length of time needed for capture and the complexity of the machinery. This research was followed up with another validation exercise looking at high-resolution 3D facial imaging of Di3D, using a full-scale plaster model, which identified a system error of 0.2 mm [26].

Earlier versions of the 3dMDface system have been reported to be one of the fastest 3D image capturing devices on the market [27]. There are only a few studies, which have validated previous versions of the 3dMDface system. Aldridge et al. [28] looked at the precision, error and repeatability associated with anthropometric landmark coordinate data collected from 3D images acquired with the 3dMDface system. The sample consisted of small children with Down syndrome or craniosynostosis. The results showed the system to be highly repeatable and precise with sub-millimeter error only.

Weinberg et al. [29] compared the earlier 3dMD system with the Genex system and manual anthropometry, assessing intra-observer precision in facial measurements between the three methods. Linear measurements were completed on head models, and although statistically significant, mean differences were found between the three methods (*P* < 0.05) the magnitude of the errors were sub-millimeter and so were not considered clinically significant. Another investigation aimed to objectively evaluate treatment outcomes in oral and maxillofacial surgery by comparing pre- and post-surgery 3D images using different registration procedures. They found the surface-based registration to be far more accurate. Furthermore, no differences were found between the different software packages [30]. A third group evaluated the handling of the system; their investigation found the system to be very reliable with a mean global error of 0.2 mm for mannequin head measurements [31].

Ghoddousi et al. [32] used human subjects to compare different methods of facial measurement by comparing manual anthropometry, 2D photographs and 3D images. They found all three methods had a good degree of repeatability, and 3D measurements compared well with manual measurements.

### Aims and objectives

Many of the previous studies looking at the 3dMD system have used the older two-pod camera system. For the purposes of this validation study, we have looked at the most recent fourth-generation system, the 3dMDface system, which is a four-pod camera system. The principal aim of this study was to assess the reliability and accuracy of this new three-dimensional imaging system (3dMDface) through comparison with manual anthropometry for system validation.

## Methods

Ethical approval was granted by King’s College London (KCL) College Research Ethics Committees (CREC).

The image capture took place in the same room in the medical photography department, using the same 3dMDface imaging system, specifically obtained for cleft patients. Preliminary surface measurements were undertaken on test objects (a tennis ball and Rubik’s cube with an affixed ruler). The image measurements on these test objects were analyzed using the same software and compared with the measuring tape, which had a resolution of 1 mm. This indicated good agreement between manual and 3D imaging data. Subsequently, six volunteer participants were recruited from the King’s College London Dental School, in accordance with the approach of Ghoddousi et al. [32], with the following exclusion criteria:

- Facial scarring
- Excess facial hair
- Severe facial asymmetry
- Facial piercing/tattoo
- Undergoing orthodontics/plastic surgery/cosmetic facial enhancement

**Fig. 1:**
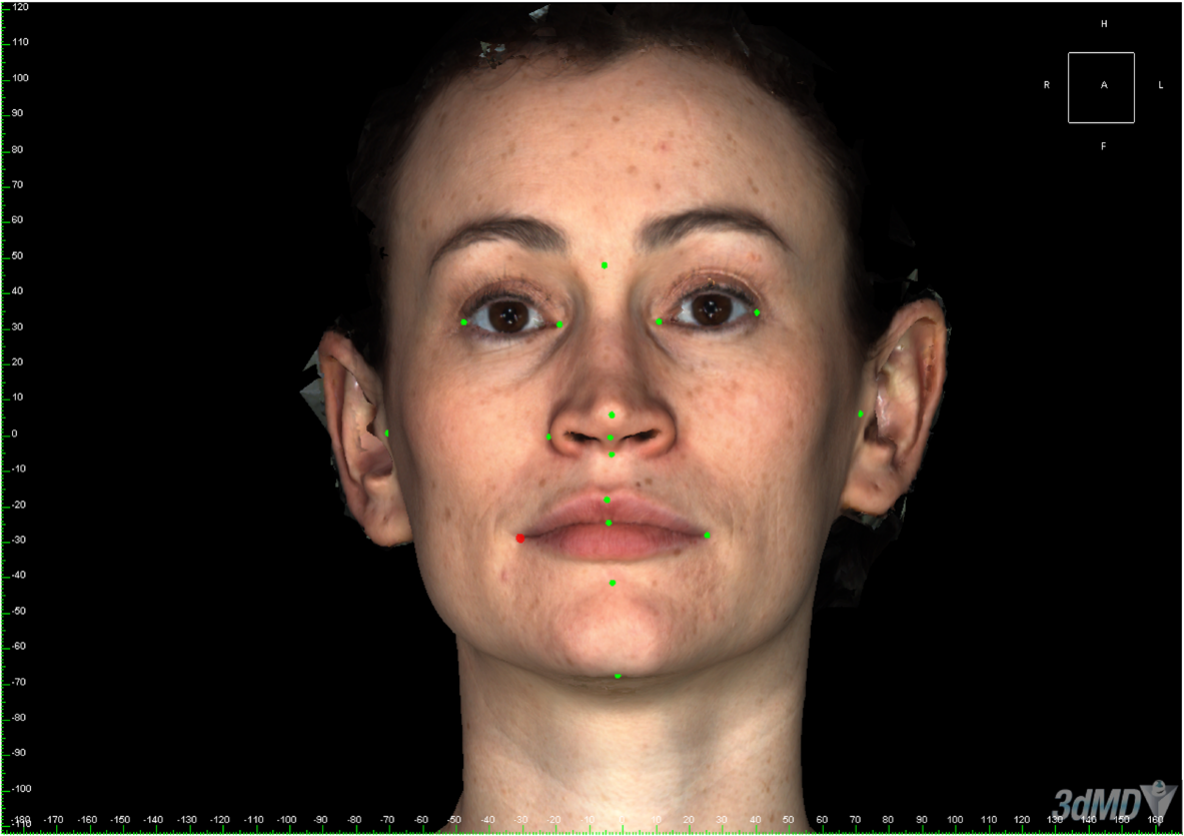
Example of a captured image; subjects were in natural head position and were asked to tie back long hair

The sample size of six subjects was based on an earlier comparable study [32]. The subjects recruited consisted of two males and four females. In addition, the six subjects have tended to be the sample size of choice in many studies of this type. The subjects were asked to wear no makeup. The subjects were positioned on a stool in front of 3dMDface imaging system and the height adjusted so that they were positioned correctly within the calibration frame, with their head in natural head position (Fig. 1). The images and measurements were taken on six different occasions, each 1 week apart, and repeated twice at each sitting. Once the image was captured, it was stored securely in the WABA (Wilde and Betts Agency) medical imaging library. They were then transferred to the 3dMDvultus software (3dMD Inc., UK) where they were analysed. The software allowed facial landmarks to be identified and inter-landmark measurements recorded (Table 1) and entered automatically on to an Excel spreadsheet. Landmark identification and placement was enhanced using the zoom in and out option and the rotation of the images. Next, manual measurements of the same facial landmarks were taken using sliding and spreading callipers and a measuring tape for surface measurement. For this part, the subject was positioned again in a relaxed fashion in natural head position sitting on the same stool. These measurements were also repeated twice at each sitting. The 3dMDface system was calibrated at the start of each session, as per manufacturer instructions. Any images that had blurring or obvious errors were deleted. All measurements, both manual and 3D, were undertaken by a single operator.

**Table 1:** Facial surface landmarks and method of measurement undertaken

Measurement	Definition	Calliper/surface measurement	Instrument used for manual measurement
Face			
Maxillary depth	sn-t	Calliper-sagittal/surface	Spreading and tape
Mandibular depth	gn-t	Calliper-sagittal/surface	Spreading and tape
Morphological face height	n-gn	Calliper-vertical	Spreading
Chin height	sl-gn	Calliper-vertical	Sliding
Orbit			
Intercanthal width	en-en	Calliper-horizontal	Sliding
Biocular width	ex-ex	Calliper-horizontal	Sliding
Nose			
Nose height	n-sn	Calliper-vertical	Sliding
Nasal tip protrusion	sn-prn	Calliper-sagittal	Sliding
Columella length	sn-c	Calliper-sagittal	Sliding
Alar surface length	ac-prn	Calliper-sagittal	Sliding and tape
Mouth			
Labial fissure width	ch-ch	Calliper-horizontal	Sliding
Upper lip height	sn-sto	Calliper-vertical	Sliding
Upper Vermillion height	ls-sto	Calliper-vertical	Sliding
Lower vermillion height	sto-li	Calliper-vertical	Sliding
Lower lip height	sto-sl	Calliper-vertical	Sliding

*ac* alar curvature point, *c* columella apex, *ch* cheilion, *en* endocanthion, *ex* exocanthion, *gn* gnathion, *li* labrale inferius, *ls* labrale superius, *n* nasion, *prn* pronasale, *sl* sublabiale, *sn* subnasale, *sto* stomion, *t* tragion (Definitions as per Naini [35])

### Statistical analysis

The 3D photographs were repeated twice at each sitting, as were the manual measurements. The data was initially entered into an Excel spreadsheet and the mean of the two measurements was found. In addition the median for each measurement on each subject over the six occasions was also calculated.

Data were tested for consistency with a normal distribution using the Ryan-Joiner test in Minitab v16 (Minitab Inc., USA). Parametric or non-parametric methods were used as appropriate, as well as the method of Bland and Altman [33] and descriptive statistics.

## Results and Discussion

Table 2 lists the median of the measurements for manual and 3D over the six visits. The manual measurements were mostly higher than the 3D measurements. This is in contrast to other comparable studies, which found the 3D measurements to be larger [32].

**Table 2:** Median manual and 3D measurements (mm)

Subject												
	A^m^	A^3D^	B^m^	B^3D^	C^m^	C^3D^	D^m^	D^3D^	E^m^	E^3D^	F^m^	F^3D^
Maxillary depth	132.11	132.80	111.26	111.20	114.71	115.62	115.93	113.74	115.91	114.27	121.23	120.84
Mandibular depth	148.00	146.17	118.92	122.87	133.83	132.79	132.54	130.01	126.43	126.71	136.37	132.71
Morphological face height	130.81	127.36	113.76	114.74	116.61	115.52	127.02	124.57	107.07	106.50	132.12	130.59
Chin height	25.38	25.98	22.05	27.59	27.47	27.61	26.34	27.36	22.71	23.15	27.39	23.00
Intercanthal width	31.65	33.59	28.80	29.49	31.82	30.65	33.35	29.70	28.30	28.19	31.31	28.88
Biocular width	101.13	97.07	93.96	88.91	91.55	88.59	89.72	84.97	88.65	86.03	92.37	89.72
Nose height	63.65	60.56	57.20	53.10	55.31	54.14	63.21	62.62	55.07	51.52	65.90	63.25
Nasal tip protrusion	21.59	21.57	25.99	20.20	18.10	17.57	20.68	19.77	22.49	22.01	20.90	21.22
Columella length	10.13	12.16	12.22	11.58	8.03	8.84	9.23	9.19	12.12	12.32	10.43	12.11
Alar surface length	39.35	38.64	30.36	29.97	31.91	31.67	32.03	30.39	31.29	30.04	36.58	38.54
Labial fissure width	52.03	48.64	50.59	45.55	59.42	56.41	48.32	46.47	50.92	48.69	55.85	53.82
Upper lip height	25.90	25.85	18.23	20.96	20.14	20.25	22.44	20.71	19.01	17.49	24.63	25.50
Upper vermilion height	12.39	12.23	7.91	8.52	8.10	7.41	7.64	6.80	7.91	6.92	10.48	9.96
Lower vermilion height	16.18	13.90	8.93	9.82	10.78	9.10	8.13	7.77	6.77	4.93	11.37	11.53
Lower lip height	22.11	21.12	16.40	16.81	16.50	16.35	17.66	16.70	17.37	16.39	25.72	24.37

Superscripts m and 3D indicate manual measurements and three-dimensional measurements, respectively

Software was written using MATLAB (The MathWorks Inc., Natick, MA, USA) in order to calculate the mean of the two measurements and compute variability using a similar approach to that previously described [32]; in summary, the range of values for each subject was divided by the median and expressed as a percentage in order to determine the measurement variability. For all measurements, except chin height and labial fissure width, there was a greater variability with the manual method compared to 3D assessment. Using a paired Wilcoxon test, overall, there was a significantly greater variability in manual compared with 3D assessments (*p* < 0.02).

The variability of measurements can be seen in Table 3. The tendency was for the manual measurements to have greater variability than the 3D measurements. In addition, both manual and 3D measurements showed higher variability for the same measurements. For the manual measurements, the greatest variability was in upper vermillion height (ls-sto; 24.87%) and columella length (sn-c; 23.73%). Similarly for the 3D measurements, the variability was greatest in the columella measurement (sn-c; 19.45%).

**Table 3:** Manual and 3D measurement variability

Variability		
Measurement	Manual	3D
Maxillary depth	2.92	2.05
Mandibular depth	3.29	2.78
Morphological face height	3.29	2.68
Chin height	8.90	12.45
Intercanthal width	7.30	4.73
Biocular width	3.47	2.10
Nose height	5.57	4.67
Nasal tip protrusion	13.29	11.35
Columella length	23.73	19.35
Alar surface length	9.24	4.54
Labial fissure width	5.47	6.37
Upper lip height	9.64	7.53
Upper vermilion height	24.87	13.04
Lower vermilion height	18.84	14.68
Lower lip height	11.48	9.84

The lowest variability in manual measurements was found to be for the maxillary depth (sn-t; 2.92%) and both mandibular depth (gn-t) and morphological face height (n-gn), which had a variability of 3.29%. The pattern seen here shows variability was less with larger measurements, potentially, as these were easy to carry out manually and the landmarks were easy to identify both manually and on the 3D image.

Table 4 shows the calculated difference between 3D and manual measurements; in addition, the absolute differences divided by the manual measurements were calculated and expressed as a percentage (% Diff) in order to assess the relative magnitude of the differences. All the percentage differences were less than 10% except for the lower vermillion height, showing a good agreement between the two methods. The overall median percentage difference was just 4%, highlighting good agreement between the two methods. The highest median difference was recorded in biocular width (ex-ex; −3.5 mm). The greatest percentage difference was seen in lower vermillion height (sto-li; 12.05%) and upper vermillion height (sn-sto; 8.10%).

**Table 4:** Differences between 3D and manual measurements

	Subject							
	A	B	C	D	E	F	Median	% Diff
Maxillary depth	0.69	−0.06	0.91	−2.19	−1.64	−0.39	−0.22	0.66
Mandibular depth	−1.82	3.95	−1.04	−2.53	0.27	−3.66	−1.43	1.57
Morphological face height	−3.45	0.99	−1.09	−2.45	−0.57	−1.53	−1.31	1.05
Chin height	0.60	5.53	0.14	1.02	0.44	−4.39	0.52	3.11
Intercanthal width	1.94	0.69	−1.17	−3.65	−0.11	−2.43	−0.64	4.90
Biocular width	−4.07	−5.05	−2.96	−4.75	−2.62	−2.65	−3.51	3.63
Nose height	−3.09	−4.10	−1.17	−0.60	−3.55	−2.65	−2.87	4.44
Nasal tip protrusion	−0.02	−5.78	−0.53	−0.91	−0.48	0.32	−0.51	2.53
Columella length	2.03	−0.64	0.81	−0.04	0.20	1.68	0.50	7.66
Alar surface length	−0.70	−0.40	−0.25	−1.64	−1.26	1.97	−0.55	2.90
Labial fissure width	−3.40	−5.04	−3.01	−1.85	−2.23	−2.03	−2.62	4.72
Upper lip height	−0.06	2.73	0.10	−1.73	−1.53	0.87	0.02	5.63
Upper vermilion height	−0.16	0.61	−0.69	−0.84	−1.00	−0.51	−0.60	8.10
Lower vermilion height	−2.28	0.90	−1.69	−0.36	−1.84	0.16	−1.02	12.05
Lower lip height	−0.99	0.41	−0.15	−0.96	−0.98	−1.35	−0.97	4.86

The smallest percentage differences were seen in the maxillary length (sn-t; 0.66%), mandibular depth (gn-t; 1.57%) and morphological face height (n-gn; 1.05%). This was in keeping with results looking at the variability.

**Fig. 2:**
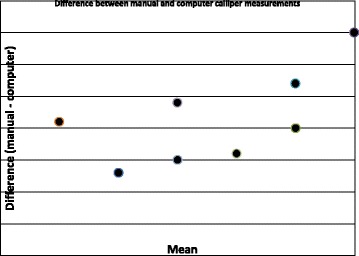
Difference between manual and computer calliper measurements

In order to compare the tape and 3D measurements, the difference was plotted against the means, demonstrated in scatter diagrams. For the calliper measurements, straight-line measurements from the Rubik’s cube and tennis ball indicated that the agreement was at least as good as the resolution of the tape (Fig. 1). However, for the surface measurements, the 3D measurements were mostly slightly smaller than the manual, for the Rubik’s cube and tennis ball (Fig. 2). Despite this distribution, the mean difference is 0.09 mm, which is less than the resolution of the tape and unlikely to be clinically relevant.

When comparing linear calliper measurements, there was no significant difference between manual and computer measurements using paired *t* test (*t* = 0.24, *p* = 0.82, *n* = 10), mean difference 0.01 mm and standard deviation 0.07. There was no significant correlation between the difference and mean of these measurements (Pearson’s correlation coefficient *r* = 0.52, *p* = 0.13).

**Fig. 3:**
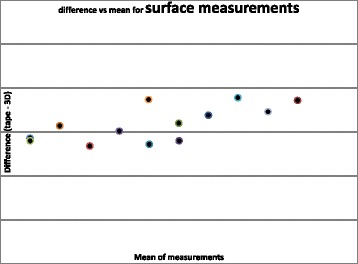
Difference vs. mean for surface measurements

Figure 2 shows a plot of the difference between tape and 3D surface measurements against mean of the measurements. When comparing the surface measurements, there was no significant difference between manual and computer measurements using a paired *t* test (*t* = −1.58, *p* = 0.14, *n* = 13), mean difference 0.09 mm (standard deviation 0.20). There was a significant correlation between the difference and mean of these measurements (Pearson’s correlation coefficient *r* = 0.69, *p* < 0.01). This implies that larger measurements tended to be associated with a higher difference (difference = −0.16 + 0.0048 mean) (see Fig. 3).

Three-dimensional imaging in cleft care provides a modern platform for recording the morphology of the facial complex. The applications of this technology extend into many surgical specialties and in the case of cleft lip and palate clinics, the technology is already being used and implemented in hospital departments internationally. The field is constantly developing and evolving with the frequent introduction of newer systems into the market. Validation exercises are important to compare whether or not the technology fares well against our current record-taking practice and its suitability for use in patients.

Investigations looking at the accuracy of newly developed 3D imaging systems have shown promising results. Lubbers et al. [31] used a phantom head model to test the precision and accuracy of the 3dMD system. They found the handling of the system to be straightforward and highly reliable with a mean global error of 0.2 mm. They found neither the position of the head nor that of the camera influenced the measurements. They recommended its use over manual anthropometry and 2D imaging. However, the use of a mannequin excludes the effects of soft tissue drape, which may considerably affect the positioning and measurement of landmarks. For this study, we used human subjects for landmarking and measurement. Ghoddousi et al. [32] implemented the use of real-life test subjects when validating a previous 3dMD system. They compared three methods of facial measurement, of which 2D photography showed the highest variability. The mean difference when comparing 3D data and manual measurement was found to be 0.23 mm (shortest distance) and 0.13 (surface).

The current study tested the latest system produced by 3dMD. Where the previous comparable studies used an older two-pod camera system, for this study, the fourth-generation 3dMDface imaging system was used. This system consisted of four camera pods, the two extra pods, added an additional superior and inferior view. Each pod housed one speckle projector and a combination of one colour (to capture colour images for surface rendering) and two monochrome cameras (to capture speckle projection); all incorporated 25-mm lenses for facial capture in addition to a flash in each pod. The results from this study highlight a high agreement between manual and 3D facial measurements, as the mean measurements derived from both were mostly similar. The larger measurements, such as maxillary length and mandibular length, presented with low variability. On the other hand, the smaller measurements (the ones more difficult to measure) found significant variability, particularly in columella length, reflecting the results of earlier comparable studies [32]. This applied to both manual and 3D measurements. The variability in the small measurements may be affected by the resolution of the measuring system.

Reasons for this variation can be related to human error in landmark identification and placement. Furthermore, the soft tissue positions and dimensions of the subject from 1 week to the next will not be stable with many variables influencing the soft-tissue dimensions. Despite this possible influencing factor, one study found the landmark variance, over time, to be as low as 0.6 mm [13]. However, it must be noted that this study completed measurements on a mannequin head model.

A more recent study looked at the accuracy and precision of a 3D anthropometric facial analysis with and without landmark labeling before image acquisition using the 3dMDface system [1]. Overall, they reported a similar accuracy between traditional anthropometry and 3D measurements, regardless of whether or not there was previous landmarking. However, it was noted that accuracy was increased with previous landmarking. Furthermore, the authors of this study found that those landmarks that required palpation for identification were associated with greater error during indirect measurement.

The subjects in this study were instructed to maintain a neutral facial expression. However, this is difficult to control and when the images were analyzed, subtle differences in expression were seen. This was found on subject A particularly in measurements around the labial fissure. This can be a result of minor facial expression. This is keeping with findings of other studies that have suggested greater error in landmarks that are difficult to see and those involving the labial fissure [34].

When analyzing the images, difficulty was found when placing landmarks on the images of subjects with darker facial complexions. Furthermore, locating the Tragion (t) was difficult due to shadowing from the subjects hair in the ear region; this problem has been reported previously [28].

The primary aim of this study was to test the validity of this technology by testing its reliability in measurements of the facial complex. The results show the 3D measurements to have less variation in comparison to manual measurements. The second aim was to identify a faster and more efficient image capture. The capturing of the image was no doubt faster than routine 2D photography; the image capture was instantaneous (1.5 milliseconds), and a single image was needed for complete facial assessment. However, at times, the set-up and calibration was found to be time consuming and re-calibration is necessary prior to capturing the image. The technology has no doubt proved to be an exceptional tool to add to the cleft care armamentarium. It provides interesting prospects for the future of patient assessment, diagnosis and treatment planning, in particular, for communication with patients, when discussing existing facial appearances and forecasting results.

For the purposes of this validation exercise, the patient was positioned in a natural head position within the calibration frame for 3D capture. The positioning of the subject was the most time consuming part of the process. Although each sitting required two images, often more were taken as incorrect positioning, or patient movement resulted in detriment to the image and artifacts. Therefore, a study to look into the effect of head position on the quality of image is crucial, with an aim to develop a simple protocol for subject positioning.

Differences in difficulties associated with landmark identification and placement on darker complexions were noted. It would be beneficial to conduct a study of a large ethnic mix, to assess the accuracy of the system with varying skin tones.

## Conclusions

- There is more variation in manual landmark identification and measurement in comparison to 3D measurements.
- The fourth-generation 3dMDface imaging system is a reliable system of facial imaging and documentation of the face.
- The 3dMDface system provides efficient and instantaneous image capture and ease of digital storage.
- The images produced are accurate and lifelike, providing a simple communication tool for patients for diagnosis and treatment planning.
